# A new heterozygous compound mutation in the *CTSA* gene in galactosialidosis

**DOI:** 10.1038/s41439-019-0054-x

**Published:** 2019-04-26

**Authors:** Hideki Nakajima, Miki Ueno, Kaori Adachi, Eiji Nanba, Aya Narita, Jun Tsukimoto, Kohji Itoh, Atushi Kawakami

**Affiliations:** 1 Department of Neurology and Strokology, Nagasaki University Hospital, 1-7-1, Sakamoto, Nagasaki, 852-8501 Japan; 2 Department of Neurology, Mita Hospital, International University of Health and Welfare, 1-4-3, Mita, Minato-City, Tokyo 108-8329 Japan; 3 Organization of Research initiative and Promotion, Tottori University, 86, Nishi-cho, Yonago, Tottori 683-8503 Japan; 4 Department of Medicinal Biotechnology, Institute for Medicinal Resources, Graduate School of Pharmaceutical Sciences, Tokushima University, 1-78, Shoumachi, Tokushima 770-8505 Japan; 5 Department of Immunology and Rheumatology, Unit of Advanced Preventive Medical Sciences, Division of Advanced Preventive Medical Sciences, Nagasaki University Graduate School of Biomedical Sciences, 1-12-4, Sakamoto, Nagasaki 852-8523 Japan

**Keywords:** Disease genetics, Genetics of the nervous system

## Abstract

Galactosialidosis is an autosomal recessive lysosomal storage disease caused by the combined deficiency of lysosomal β-galactosidase and neuraminidase due to a defect in the protective protein/cathepsin A. Patients present with various clinical manifestations and are classified into three types according to the age of onset: the early infantile type, the late infantile type, and the juvenile/adult type. We report a Japanese female case of juvenile/adult type galactosialidosis. Clinically, she presented with short stature, coarse facies, angiokeratoma, remarkable action myoclonus, and cerebellar ataxia. The patient was diagnosed with galactosialidosis with confirmation of impaired β-galactosidase and neuraminidase function in cultured skin fibroblasts. Sanger sequencing for CTSA identified a compound heterozygous mutation consisting of NM_00308.3(CTSA):c.746 + 3A>G and c.655-1G>A. Additional analysis of her mother’s DNA sequence indicated that the former mutation originated from her mother, and therefore the latter was estimated to be from the father or was a de novo mutation. Both mutations are considered pathogenic owing to possible splicing abnormalities. One of them (c.655-1G>A) is novel because it has never been reported previously.

Galactosialidosis (GS, OMIM #256540) is an autosomal recessive lysosomal storage disorder (LSD) caused by a primary defect of protective protein/cathepsin A (PPCA) and/or a secondary defect of components of the lysosomal multienzyme complex (LMC), which includes the two glycosidases, β-galactosidase (β-Gal) and neuraminidase-1 (NEU1)^1,2^. PPCA is also designated as cathepsin A (CTSA) and is one of the serine carboxypeptidase-type enzymes that protects and stabilizes the LMC from lysosomal degradation^3^. Until now, 35 mutations in the *CTSA* gene in patients with GS have been published (Supplemental Table). In these patients, almost all mutations belonged to the early infantile and late infantile types. Most juvenile/adult type cases are of Japanese origin^4,5^. Although the majority of GS cases belong to the juvenile/adult type, there are very few case reports with a mutation, let alone heterozygous mutations. Patients with juvenile/adult type GS present broad clinical symptoms: coarse facies, vertebral changes, cherry-red spots and neurological complications, such as myoclonus, cerebellar ataxia, epilepsy, and cognitive impairment. In general, hepatosplenomegaly and angiokeratoma, typical manifestations in GS, are often absent in juvenile/adult type cases. Even though the prognosis is not as poor as those of the early infantile and late infantile types^6,7^, there is no definitive treatment.

A 23-year-old Japanese female was admitted for hand tremors and unsteadiness on her feet. Her parents were nonconsanguineous. Though she had been admitted to a pediatric clinic to investigate the cause of her short stature when she was 10 years old, no examinations indicated hormonal abnormalities. Her mother had no abnormal findings except for her short stature, and the patient could not contact her father after her parents’ divorce. From the age twenty, she presented with ataxia while walking and had hand tremors. She had coarse facies (Fig. 1a), short stature (135 cm) and angiokeratoma (Fig. 1b) on her extremities. Neurologically, she presented no weakness of muscles and had normal deep tendon reflex but had action myoclonus of the hands and cerebellar ataxia; her scanning speech, nose-to-finger and heel-to-knee tests were clumsy.

**Fig. 1:**
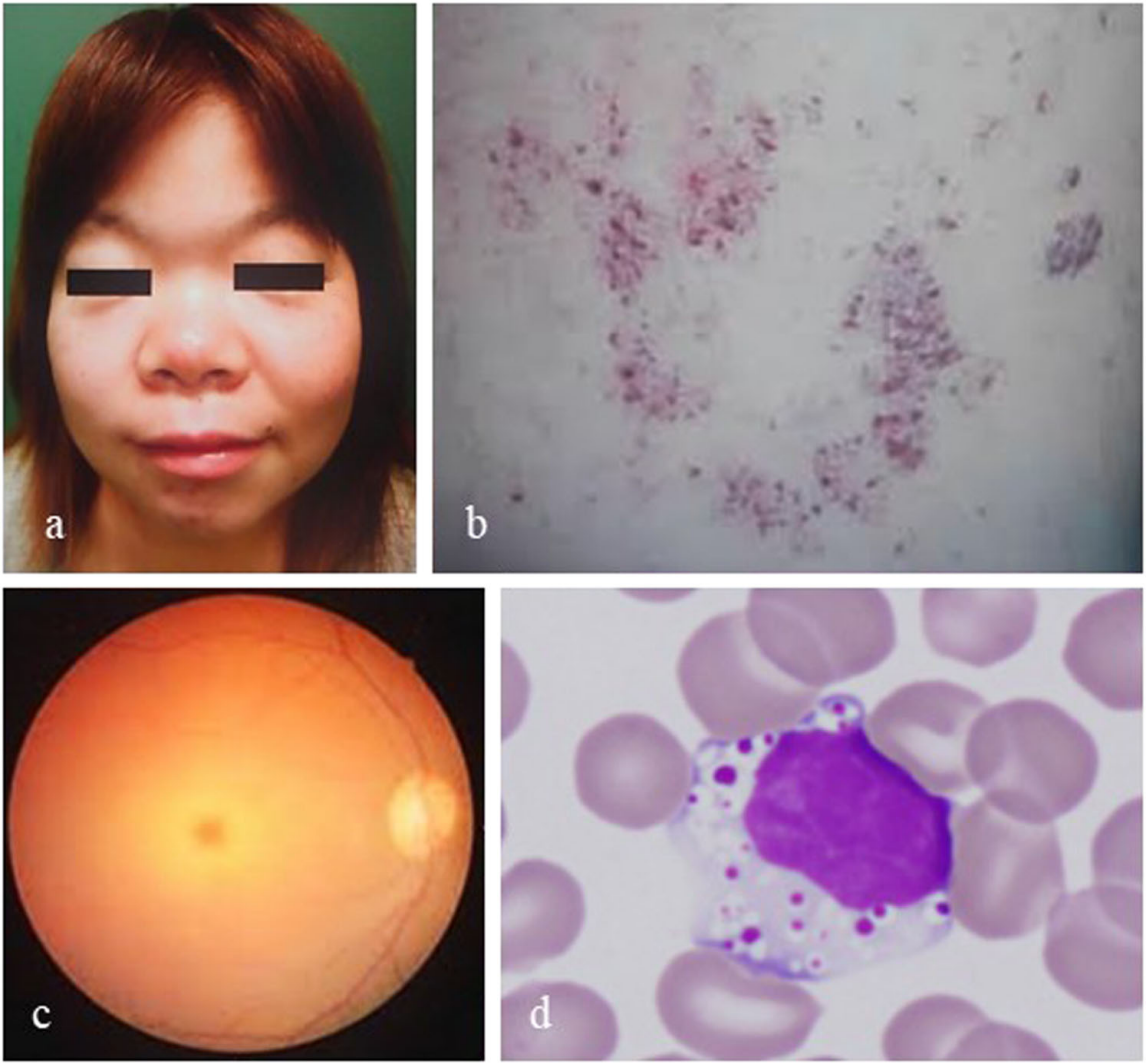
Characteristic findings of our patient. **a** Facial features: coarse facies, large nose, depressed nasal bridge, hypertelorism, and micrognathia. **b** Angiokeratoma of arm skin. **c** A cherry-red spot of the right eye ground in funduscopy. **d** Peripheral blood smear with May-Giemsa stain shows vacuolation of a lymphocyte

Neuropsychological assessments indicated that her Wechsler adult intelligence scale (WAIS)-III intelligence quotient (IQ) was 64 (verbal IQ 83, performance IQ 52), and the Mini-Mental State Examination was 27/30. An ophthalmologist found a cherry-red spot on her eye ground (Fig. 1c). There was no abnormal data in general blood and biochemistry tests, but vacuolation of lymphocytes (Fig. 1d) was observed. X-rays showed thoracic and vertebral deformities, computed tomography scan showed mild hepatosplenomegaly, and brain magnetic resonance image displayed mild atrophy of cerebral and cerebellar cortexes for her age (data not shown). Echocardiography demonstrated moderate aortic and mitral valve regurgitations. Electroencephalography did not show any giant somatosensory evoked potential or C-reflex.

Because of the cherry-red spot on her eye ground and vacuolation of lymphocytes, we suspected that she had a type of LSD. We tested urine catabolites but could not find any accumulation of uronic acids. Next, we analyzed the levels of 10 lysosomal enzymes: β-gal, α-galactosidase, β-glucosidase, α-glucosidase, β-hexosaminidase A, α-mannosidase, α-fucosidase, β-glucuronidase, and NEU. The levels of β-gal and NEU in cultured skin fibroblasts were 111.2 nmol/mg protein/h (normal 401 ± 184.8) and 0 (normal 25.0 ± 17.0), respectively, but those of the other 8 enzymes were in the normal range. She was diagnosed with GS. Next, CTSA gene analysis was performed by using the Sanger method, and a compound heterozygous mutation was identified; NM_00308.3(CTSA):c.746 + 3A>G and c.655-1G>A (Fig. 2). c.746 + 3A>G, which is considered to cause splicing abnormalities, is a common variant in Japanese GS patients as a homozygous pattern^8,9^. Subsequent analysis for the sample from the mother identified the c.746 + 3A>G variant. Thus, this variant is confirmed to be inherited from the mother. On the other hand, c.655-1G>A has never been reported previously, indicating a novel variant. Because we could not obtain a sample from the father, the origin of the c.655-1G>A variant is unknown.

**Fig. 2:**
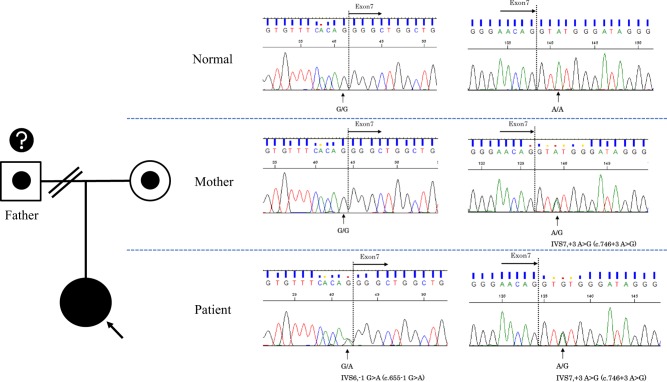
Direct sequencing of *CTSA* gene exon 7 from normal control (upper), the patient (middle), and her mother (lower). IVS7,+3A>G (c.746 + 3A>G) was inherited from the mother^8,9^. Neither we nor the patient could contact her father

After diagnosis, we prescribed carbamazepine and clonazepam, and her action myoclonus improved, but ataxia remained as before. After this admission, we additionally tested the levels of residual CTSA (acid carboxypeptidase) activity: 37.7 nmol/mg protein/h (control 2,796). We confirmed CTSA deficiency in cultured skin fibroblasts.

Reports about the mutations in *CTSA*, except in the CpG islands^10^, have been relatively few even though the *CTSA* gene is approximately 43,000 kb, includes 15 exons and is thus larger than other human genes. The reason is not clear, but difficulty in diagnosing GS clinically seems to be relevant.

For the stability of LMC, CTSA plays a role as a molecular chaperone protein. Then, CTSA matures and stabilizes β-Gal with processing of its carboxy terminus and promotes oligomerization and activation of NEU1^6,11,12^. In GS patients, sialylated oligosaccharides, and glycopeptides accumulate in lysosomes of every tissue, which causes various clinical manifestations, as in this case. The patient presented with ataxia, which was presumably caused by decreased cerebellar Purkinje cells^13,14^, and the myoclonus was caused by a disturbance in the sensorimotor cortex by way of the thalamic tract or cerebellothalamic tract^15^ because of the abnormal accumulation of sialylated oligosaccharides and glycopeptides. The magnetic resonance image of the patient also indicated mild atrophy of the cerebellar and cerebral cortexes for her age.

Normally, CTSA acts as a serine carboxypeptidase, deaminase, and esterase in the processing of bioactive peptides substance P, oxytocin, and endothelin^2,16^ and in the formation of elastic fibers on the cell membrane^17^. Therefore, mutation of *CTSA* produces a variety of complications in endothelial cells, heart, kidney, central nervous system, and so on. The identified compound heterozygous mutation NM_00308.3(CTSA):c.746 + 3A>G & c.655-1G>A might be related to the remarkable neurological manifestations the patient presented. The c.746 + 3A>G variant has been reported by Shimmoto et al.^9^ with juvenile/adult type cases of exon 7 skipping^9^, and the single-nucleotide variant was evaluated as pathogenic by the Clin Var and HGMD^®^ databases. c.655-1G>A is also located next to the canonical splice site: consequently, it might cause some aberrant splicing of exon 7. According to the ACMG standards and guidelines^18^, the c.655-1G>A is also considered pathogenic, although functional studies have not been performed. By analyzing the patient’s mother’s DNA sequence, we could confirm that c.746 + 3A>G originated from the mother; therefore, we determined that c.655-1G>A was from the father or was a de novo variant. A certain study reported that the amounts of residual acid carboxypeptidase activities did not correlate with clinical phenotypes, but the enzyme deficiency is closely connected to the genetic defect of protective protein, CTSA^19^. Therefore, it is possible that the IVS7, +3A>G mutation of the *CTSA* gene is one of the contributing factors to her short stature because the mother was only a carrier without other complications.

Some limitations exist in this study. First, we were not able to confirm the heterozygosity with the father’s sample because he was unable to be located. Second, owing to the sample condition, we did not obtain scientific evidence that confirmed splicing abnormalities are caused by c.655-1G>A. These problems will be our next research topics.

We, as clinicians should observe each symptom carefully and treat it with the best supportive care. This compound heterozygous mutation in the *CTSA* gene would also be a new target for enzyme replacement therapies and gene therapies such as bone marrow transplantation using transgenic bone marrow cells overexpressing the corrective enzymes^14^ or gene delivery with a recombinant vector^20^. We hope these findings will contribute to the elucidation of the pathogenesis and treatment of GS.

## Supplementary information

Supplementary Table

## Data Availability

The relevant data from this Data Report are hosted at the Human Genome Variation Database at 10.6084/m9.figshare.hgv.2573.
