# Supplementary material for: A new heterozygous compound mutation in the CTSA gene in galactosialidosis
Source: Hum Genome Var. 2019 Apr 26;6:22. doi: 10.1038/s41439-019-0054-x (PMC6486599; doi:10.1038/s41439-019-0054-x)
Supplement: Supplementary file 1 — Supplementary Table [file 41439_2019_54_MOESM1_ESM.doc]

**Supplemental table**　Identified CTSA mutations in galactosialidosis patients

| Case | Codon number | Exon | Phenotype | DNA / protein change | Type of mutations | Authors, year |
| --- | --- | --- | --- | --- | --- | --- |
| 1 | 37 | － | Unknown | c.108, 110delGCT / p.Lys37del | Deletion | Malvagia S et al, 2004 |
| 2 | － | － | Unknown | c.60delG | Deletion | Malvagia S et al, 2004 |
| 3 | 67 | 2 | EI | p.Gln67Arg | Missense | Shimmoto M et al, 1990 |
| 4 | 69 | 2 | EI | p.Ser69Tyr | Missense | Zhou XY et al, 1996 |
| 5 | 83 | 2 | EI | p.Val83Arg | Missense | Shimmoto M et al, 1993 |
| 6 | 95 | － | － | c.284delC | Deletion | Unidad de Diagnostico y Tratamiento de Errores Congenitos del Metabolismo |
| 7 | 103 | 3 | EI | p.Gly103Ser | Missense | Groener J et al, 2003 |
| 8 | 103 | 3 | EI | p.Gly103Val | Missense | Kiss A et al, 2008 |
| 9 | 108 | 3 | EI | p.Ser108Leu | Base substitution | Shimmoto M et al, 1990 |
| 10 | 116 | 3 | EI | p.His116Arg | Missense | Catiotti A et al, 2013 |
| 11 | 150 | 5 | EI | c.448A>G / p.Val150Met | Missense | Zhou XY et al, 1996 |
| 12 | 189 | － | － | c.546delTT | Deletion | Richard C et al, 1998 |
| 13 | 191 | － | LI | c.571_572delTT / p.Phe191Profs | Deletion, | Richard C et al, 1998 |
| 14 | 217 | － | － | c.649delC | Deletion | Shamseldin HE et al, 2015 |
| 15 | 236 | － | EI | c.707T>C / p.Leu236Pro | Missense | Zhou XY et al, 1996 |
| 16 | 254 | 8 | EI | p.Leu254Pro | Missense | Zhou XY et al, 1996 |
| 17 | 259 | 8 | EI | p.Cys259Arg | Missense | Catiotti A et al, 2013 |
| 18 | 267 | 8 | LI | p.Tyr267Asn | Missense | Zhou XY et al, 1996 |
| 19 | 296 | － | － | c.887_888delAT | Deletion | Zhou XY et al, 1996 |
| 20 | 347 | － | － | c.904insC | Insertion | Groener J et al, 2003 |
| 21 | 349 | － | EI | c.1045T>A / p.Cys349Ser | Missense | Kostadinov S et al, 2014 |
| 22 | 406 | － | － | p.406Gln* | Nonsense | Catiotti A et al, 2013 |
| 23 | 413 | 13 | EI | p.Tyr413Cys | Missense | Shimmoto M et al, 1990 |
| 24 | 424 | 13 | EI/LI | c.1271T>C / p.Met424Tyr | Missense | Zhou XY et al, 1996 |
| 25 | 441 | － | EI | c.1321C>T / p.Arg441Cys | Missense | Kostadinov S et al, 2014 |
| 26 | 442 | 14 | LI | p.Arg442Trp | Missense | Kiss A et al, 2008 |
| 27 | 457 | 14 | EI | c.1369G>A / p.Gly457Ser | Missense | Zhou XY et al, 1996 |
| 28 | 458 | 14 | LI | c.1372T>G / p.Phe458Val | Missense | Zhou XY et al, 1991 |
| 29 | 471 | 14 | LI | c.1411A>G / p.Lys471Glu | Missense | Takiguchi K et al, 2000 |
| 30 | 475 | 15 | LI | c.1424A>C / p.His475Pro | Missense | Garcia Hernandez L et al, 2018 |
| 31 | － | － | EI | c.1284delG / p.Leu34Afs | Deletion | Kartal A et al, 2017 |
| 32 | － | － | Unknown | IVS2 +1G>T | Splicing | Malvagia S, et al, 2004 |
| 33 | － | － | Unknown | IVS3 ds+1G>T | Splicing | Malvagia S, et al, 2004 |
| 34 | － | Intron 7 | J/A | c.746+3A>G, IVS7 ds+3A>G | Splicing | Shimmoto M et al, 1990 |
| 35 | － | － | L/I | IVS8 ds+9C>G | Splicing | Richard C et al, 1998 |
| This report | － | － | J/A | c.655-1G>A , IVS6, -1G>A | Splicing? | Nakajima et al, 2019 |

Abbreviations: EI early infantile, LI late infantile, J/A juvenile adult.
